# The Phenolic Acid Content in Wheat Depending on the Intensification of Cultivation Technology

**DOI:** 10.3390/foods14040633

**Published:** 2025-02-13

**Authors:** Leszek Rachoń, Tomasz Cebulak, Barbara Krochmal-Marczak, Ireneusz Kapusta, Izabela Betlej, Anna Kiełtyka-Dadasiewicz

**Affiliations:** 1 Department of Plant Production Technology and Commodity Science, University of Life Sciences in Lublin, Akademicka 13, 20-950 Lublin, Poland; leszek.rachon@up.lublin.pl; 2 Department of Food Technology and Human Nutrition, Institute of Food Technology and Nutrition, College of Natural Sciences, University of Rzeszów, 35-601 Rzeszów, Poland; tcebulak@ur.edu.pl (T.C.); ikapusta@ur.edu.pl (I.K.); 3 Department of Plant Production and Food Safety, University College of Applied Sciences in Krosno, 38-400 Krosno, Poland; barbara.marczak@pans.krosno.pl; 4 Institute of Wood Sciences and Furniture, Warsaw University of Life Sciences SGGW, 159 Nowoursynowska St., 02-776 Warsaw, Poland; izabela_betlej@sggw.edu.pl

**Keywords:** common wheat, spelt, durum, einkorn, phenolic acid

## Abstract

Phenolic acids were identified, and their content was determined in the grain of four species of wheat: common wheat (*Triticum aestivum* ssp. *vulgare*), spelt (*T. aestivum* ssp. *spelta*), durum (*T. turgidum* ssp. *durum*), and einkorn (*T. monococcum*) grown at two different levels of cultivation technology: medium and high. Thirteen acids were identified for each species. Einkorn cultivar PL 5003 had the highest content of phenolic acids, reaching up to 2106 mg 100 g^−1^ DM. The response of various species to the levels of technology applied was varied, but ferulic acid was always predominant (465–868 mg 100 g^−1^ DM). Common wheat and spelt responded with a decrease in the content of the acids when tested using the higher level of technology (on average by 265 and 62 mg), while their content increased in durum wheat and einkorn (282 and 352 mg). A clear response to weather conditions was also observed; most of the genotypes had a higher content of phenolic acids when there was more rainfall. The present study provides the basis for using these genotypes to produce food with increased nutritional content using appropriate agricultural procedures.

## 1. Introduction

Cereal grain and its products have accompanied human civilisation since its beginnings as one of the main pillars of the diet [1,2,3,4]. However, the industrial revolution at the turn of the 20th century led to the emergence of a new dietary model. In particular, after World War II, highly processed food products, often refined, began to dominate, and nutritional value consisted mainly of energy components such as proteins, fats, and carbohydrates, while health-promoting substances were overlooked. The development of effective and rapid food production methods has directed attention to wheat as a prolific cereal from which bread is quickly produced [5]. The attention of food producers and processors has mainly been directed towards cultivars producing high yields with a high gluten content.

At the start of the 1970s, an increase in chronic non-communicable diseases resulting from poor nutrition began to be observed. As a result, more significant research was placed on the impact of biologically active substances naturally present in raw plant materials. At this time, attention also began to be paid to cereal cultivars with a better profile of bioactive compounds. Such compounds include polyphenols, particularly phenolic acids (PAs), the largest and most common group of antioxidant compounds in the grains of cereal plants [6]. The most abundant PA in wheat is ferulic acid, the concentration of which in the grain is not associated with the means of production but rather with climate factors during the growing period of the species [7]. PAs are derivatives of benzoic and cinnamic acids present in all cereals. Pas exist in cereals in both free and bound forms [8,9]. Most free PAs are found in the outer layers of the seed coat and embryo. These compounds are mainly bound to carbohydrate fractions in wheat grain, and their availability is highest in the large intestine following fermentation and enzymatic extraction [10]. PA has multiple functions in cereals. They act as stabilisers of the cell wall structure but can also be involved in wheat’s physical and chemical defense against various microorganisms, pests, and insects [9,11].

A World Health Organization report for 2012–2016 [12] suggests that the consumption of whole-grain products can reduce the risk of chronic non-communicable diseases associated with increased oxidative stress (e.g., type 2 diabetes, cardiovascular diseases, hypertension, and obesity). The cause of the increase in metabolic diseases is an unhealthy lifestyle and a lack of fibre and bioactive compounds in the diet. Wholemeal products have significant biological activity associated with PAs in the bran and aleurone layer [13,14,15]. A significant reduction in pro-inflammatory cytokines supports the anti-inflammatory activity of phenolic compounds in humans. Food produced from whole wheat grain has a higher ferulic and dihydro ferulic acid content than refined grain products [16].

Due to growing nutritional awareness, consumers are becoming interested in old, alternative cereal cultivars with better health-promoting value [3,4], essential to preventing or treating cancer, cardiovascular disease, atherosclerosis, and neurodegenerative disorders. Dietary supplementation with extracts rich in phenolic compounds helps with the effective management of these disorders [17]. Following digestion, insoluble forms of PA are transformed in the human gastrointestinal tract, where through the activity of enzymes of the microbiota, PAs are released, increasing their bioavailability [18]. Among the many species of food cereals, the highest content of PA compounds has been shown in maize, followed by wheat [19]. PAs exert antibacterial effects and show potential as a preservative in food and food packaging materials. The consumption of whole-grain products is conducive to synthesising short-chain fatty acids, which are an excellent substrate for beneficial intestinal microflora and, therefore, can prevent insulin resistance and reduce the risk of colorectal cancer [9,20,21]. A diet rich in PAs can have a qualitative and quantitative effect on the intestinal microbiota, thereby inducing indirect health consequences in mammals through the action of these microorganisms. Moreover, PAs can be fermented by the intestinal microbiota, which modulates the bioactivity of these compounds. In the colon, PAs exhibit anti-inflammatory, antioxidant, and antiproliferative activity [22]. Santos et al. [23] confirmed that the content of PAs depends on the genotype of common wheat and the degree of ripeness in the seeds. According to Yilmaz et al. [14], the phytochemical composition of wheat is modified by several factors, including genetic origin, cultivation technology, and climate conditions.

In consideration of the importance of PAs in the human diet and, at the same time, the variation in their content in wheat grain, we carried out a study aimed at identifying PAs and determining their content in the grain of four genotypes of wheat grown as winter crops at two levels of cultivation technology: medium and high.

## 2. Materials and Methods

### 2.1. Plant Material and Field Experiment

The material used for this study was the grain of four species of winter wheat: common wheat (*Triticum aestivum* ssp*. vulgare*)—‘Tonacja’ cultivar; durum wheat (*T. turgidum* ssp. *durum*)—‘Komnata’; spelt *(Triticum aestivum* ssp. *spelta*—‘Schwabenkorn’; and einkorn wheat *T. monococcum*—PL 5003. A list of the *Triticum* species and cultivars tested in this study is presented in Table 1.

The wheat was grown using two different levels of cultivation technology: a medium level (A), with the application of mineral fertilisers (N 70, P 30.5 and K 99.6 kg ha^−1^), seed dressing, and weed control and a high level (B), with increased nitrogen application (N 140, P 305 and K 99.6 kg ha^−1^), seed dressing, weed control, two treatments against disease, an insecticide, and a growth regulator. The field experiment was conducted in 2011/2012 and 2012/2013 on the Felin Experimental Farm belonging to the University of Life Sciences in Lublin, Poland (51°22′ N, 22°64′ E). The experiment was set up on soil classified as having a good wheat complex, valuation class 2. The soil had a neutral pH of 6.6, high content of phosphorus (186 mg P_2_O_5_ kg^–1^ soil), average content of potassium (155 mg K_2_O kg^–1^ soil), and low content of magnesium (47 mg Mg kg^–1^ soil) [24]. The precursor crop was winter rapeseed. Post-harvest treatments were carried out after harvesting the precursor crop, and Good Agricultural Practice applied phosphorus and potassium fertilisers. The area of the plots for sowing was 22 m^2^, and the area for harvest was 10 m^2^. Wheat was sown at 500 seeds per m^2^. The weather conditions for the crop are presented in Table 2. Wheat was harvested at the fully ripe grain stage (BBCH 89) using a plot combine. After harvest, the grain was cleaned, and the moisture content was adjusted to 14%.

### 2.2. Weather Conditions

Analysis of the weather conditions in the years of the study shows substantial differences in average air temperatures and rainfall totals compared to the long-term average (Table 2). Moisture conditions in the autumn of 2011 were unfavourable to the germination and emergence of winter wheat (rainfall totals in September and October were much lower than the long-term averages). Temperatures were higher than the long-term averages, meaning that the winter forms of wheat entered winter dormancy, producing many tillers. The uniform rainfall distribution in the spring of 2012, during intensive cereal growth (April–May), supported by high temperatures, was favourable to wheat development and high yields. The rainfall totals from April to July were higher than the long-term averages. The relatively dry August enabled timely harvest and good grain quality. The year 2013 was favourable for winter forms regarding moisture, with more abundant rainfall, especially in May and June, which form the critical stage for cereals. Rainfall totals from April to July were higher than the long-term averages. The relatively dry August enabled timely harvest and good grain quality.

### 2.3. Extraction Procedure

The wheat grain was ground using a LabMill mill (Perten, Hägersten, Sweden) equipped with a homogenising sieve with a d = 0.5 mm mesh size. The flour was freeze-dried for 48 h in an Alpha 1–2 LD plus laboratory freeze dryer (Martin Christ Gefriertrocknungsanlagen GmbH, Osterode am Harz, Germany) and then ground into particles ≤0.5 mm in diameter. The first extraction step involved basic hydrolysis of a 2 g sample in a 4 M NaOH solution, with boiling temperature maintained for 2 h. Phenolic acids (PAs) were extracted from the hydrolysates by the method described by Mpofu et al. [25] and modified according to Żuchowski et al. [26] and Kaszuba et al. [27]. The extraction was performed in triplicate. The validation of the results was compared to that of an internal chromatogram standard.

### 2.4. Determination of Polyphenolic Compounds

#### 2.4.1. Extraction and Separation of Phenolic Acids

The tested wheat grain was ground in a LabMill mill (Perten, Hägersten, Sweden) equipped with a homogenising sieve with a d = 0.5 mm mesh diameter. In the first extraction stage, alkaline hydrolysis of a 2 g sample was carried out in a 4 M NaOH solution, maintaining a 100 °C temperature for two hours. PA was extracted from the hydrolysates using the method of Mpofu et al. [25] with modification according to Żuchowski et al. [26]. The extraction was carried out three times. Phenolic acid concentration profiles were obtained by UPLC-PDA-MS/MS using a Waters ACQUITY UPLC liquid chromatograph (Waters Corporation, Milford, MA, USA) equipped with a photodiode array (PDA) detector coupled to a tandem quadrupole mass spectrometer (Waters ACQUITY^®^ TQD (Tandem Quadrupole Detector), Micromass, Wilmslow, UK). The following parameters were used for the TQD: a capillary voltage of 3.5 kV; a condensation voltage of 30 V in negative mode; a source temperature maintained at 250 °C and a desolvation temperature at 350 °C; a condensation gas flow of 100 L/h; and desolvation gas flow of 800 L/h. Argon was used as the collision gas at a 0.3 mL/min flow rate. Samples were separated at 50 °C on a Waters ACQUITY UPLC^®^ HSS C18 column (2.1 × 100 mm, 1.8 μm). The mobile phase consisted of eluent A (0.1% formic acid in water, *v*/*v*) and eluent B (0.1% formic acid in acetonitrile, *v*/*v*). The solvent gradient was programmed as follows: 5 min, 0% B; 0.5 min, 1% B; 2.5 min, 10% B; 10 min, 10–100% B; 1 min, 100% B; 1 min, 100–0% B. The flow rate was maintained at 0.3 mL/min, and the injection volume was 5 μL. All determinations were performed in triplicate. The method was validated for parameters such as linearity, accuracy (relative error, RE), limit of detection (LOD), limit of quantification (LOQ), and precision (relative standard deviation, RSD). Quantification was determined by external standard calibration. Stock standard solutions of the polyphenols were prepared with methanol. Each standard’s calibrator was prepared by the dilution of stock solutions, and the calibration curve was generated by plotting the peak area ratio of phenolic acid versus the nominal concentration ranging from 0.05 to 50 µg mL^−1^ (R^2^ 0.999). The regression equation was obtained by weighted (1/c2) least-squares linear regression. The LOD had a signal-to-noise ratio (S/N) of 3:1, and the LOQ had an S/N of >10. An acceptable RE within 20% and an RSD not exceeding 20% should be obtained.

#### 2.4.2. Phenolic Acid Identification

PA identification was based on the analysis of phenolic acid standards (4-OH-benzoic, caffeic, 3-OH benzoic, syringic, *p-*coumaric, *o*-coumaric, ferulic, sinapic) obtained by chromatographic analysis. Specific maximum UV absorbance spectra, mass-to-charge ratio (*m*/*z*), and fragmentation spectra resulting from collision-induced dissociation (CID) were compared. Diferulic acid isomers (1–5) were identified based on CID spectra.

#### 2.4.3. Quantitative Analysis of Phenolic Acids

A Multiple Reaction Monitoring (MRM) experiment was used for quantitative analysis, in which the presence of fragment ions originating from the selected precursor was monitored. The phenolic acid content was calculated based on the calibration curves of the dependence of the peak area on the concentration of the substance injected into the column. The PA content in wheat grain was converted to DM content and reported as the mean values from three replicates. The PA concentration in the samples was calculated from standard curves by injecting solutions with known concentrations ranging from 0.05 to 5 mg/mL (R^2^ ≤ 0.9998) of phenolic acid as standards.

### 2.5. Statistical Analysis

Results were presented as the mean of three independent results. All statistical analyses were performed using Statistica software version 13.3 (StatSoft, Krakow, Poland). The significance of the means was tested using Duncan’s post hoc test, an element of one-way analysis of variance (ANOVA). Differences at *p* < 0.05 were considered significant. Scaled heat maps prepared in R studio (Version 2024.12.1+563) were used to describe the relationships between the analysed variables.

## 3. Results and Discussion

### 3.1. Identification of Phenolic Acids (PAs) in Winter Wheat Grain

Table 3 presents the profile of PAs identified by LC-MS/MS in the grain of four winter wheat genotypes. In total, 13 PAs were identified. Kaszuba et al. [27] identified a similar number of PAs in wheat grain, while Laddomada et al. [28], in a study of genetic variation in the concentrations of PA in tetraploid wheat (*Triticum turgidum* L.), identified fewer PAs. In the present study, PA was represented by peaks 1–13, including three hydroxybenzoic acids (peaks 1, 3, and 4) and 10 hydroxycinnamic acids (peaks 2, 5–13). ESI-MS signals at *m*/*z* 137 (peaks 1 and 3) and *m*/*z* 197 (peak 4) were identified as 4-hydroxybenzoic acid, 3-hydroxybenzoic acid, and syringic acid, respectively, by comparing their retention times and mass spectra with data from an authentic standard. Following the fragmentation by MS/MS, both 4-hydroxy- and 3-hydroxybenzoic acids produced ions at *m*/*z* 93 due to the loss of CO_2_ from the precursor ions. This fragmentation pattern is a characteristic feature of hydroxybenzoic acid derivatives, as in the case of other PAs. On the other hand, syringic acid first lost a water molecule, generating the main ion at *m*/*z* 179, and then lost carbon dioxide, generating a second fragment at *m*/*z* 135. Five more PAs were identified in our study: caffeic, *p*-coumaric, *o*-coumaric, ferulic, and sinapic acid. These were identified by comparing their retention times and characteristic mass spectra with data for authentic standards. Pseudomolecular ions of *p*-coumaric acid (*m*/*z* 163), *o*-coumaric acid (*m*/*z* 163), and ferulic acid (*m*/*z* 193) produced the primary fragment ions at *m*/*z* 119, 119, and 149, respectively, but in the case of ferulic acid, carbon dioxide was lost, resulting in a second fragment at *m*/*z* 134. Another fragment generated during MS/MS was sinapic acid at *m*/*z* 223. Following fragmentation by MS/MS, sinapic acid generated ions at *m*/*z* 179 due to the loss of CO_2_ from the precursor ions. In the case of ferulic acid, carbon dioxide was lost, resulting in a second fragment at *m*/*z* 149. The other hydroxycinnamic acid derivative was caffeic acid and was identified based on precise mass measurements and MS/MS spectral data. The preliminary mass spectrum for caffeic acid showed the deprotonated molecule [M − H]^−^ ion at *m*/*z* 179. The primary fragment ions obtained in MS/MS analysis were *m*/*z* 161 and 135, corresponding to the loss of water and carbon dioxide molecules. Five isomers of di-ferulic acid (9–13) were also identified in the study. These compounds produced primary fragment ions for all of them: 297, 245, 319, 193, and 293, respectively. A similar fragmentation of PA has been described by Kaszuba et al. [27] in the grain of selected Polish cultivars of triticale and processed triticale products.

### 3.2. Total Content of PA in the Grain of Winter Wheat Genotypes Depends on the Cultivation Technology Level

The total content of PA in the grain of the winter wheat cultivars grown in the 2011/2012 and 2012/2013 growing seasons at different levels of cultivation technology (A and B) are presented in Table 4. The statistical analysis showed that the level of PA in the grain of winter wheat varied and depended on the genotype, weather conditions during the growing period, and level of cultivation technology. The total content of PA in the grain ranged from 903.21 mg 100 g^−1^ to 2106.06 mg 100 g^−1^ (Table 4). In a study by Martini et al. [29], the content of PA averaged 1499.1 ± 230.9 mg/kg DW and ranged from 1190.1 to 2052.3 mg/kg DW. These values are lower than those obtained in the present study.

On the other hand, Bellato et al. [2] reported PA levels ranging from 98.6 to 144.9 mg/kg DW in 19 cultivars of *T. durum* wheat. In another study, Menga et al. [30], in 30 genotypes of *T. durum* wheat, obtained an average PA content of 882 mg/kg DW. According to Martini et al. [29] (2015), differences in the content of PA may be due to different extraction methods. However, Martini et al. [29] and Menga et al. [30] reported that the content of PA in wheat grain depended most on climate and soil conditions. Barański et al. [31] confirmed that the content of individual PA depended on the weather conditions during the wheat cultivation years, but this relationship varied depending on the species.

Among the PAs identified, ferulic acid was predominant (Table 4). According to Mattila et al. [32] and Guo and Beta [16], ferulic acid is present in wheat grain in the most significant amounts, which agrees with our findings. However, the content of this compound in our study varied between the years of the study, the species, and the level of cultivation technology. The highest content of ferulic acid, amounting to 1017 mg of 100 g^−1^ DW, was obtained in the grain of *T. monococcum*, cultivar PL 5003, harvested in 2013 from cultivation technology B, distinguished in part by a high level of nitrogen application. The lowest ferulic acid content, 465.2 mg of 100 g^−1^ DW, was obtained in the grain of *T. tugidum* ssp*. durum* wheat, a ‘Komnata’ cultivar, which was harvested in 2012 from cultivation technology A, with average nitrogen application. Zuchowski et al. [33] (2011), in their study on the content of PA in spring and winter wheat cultivated using organic and conventional methods, reported that the predominant PA in the grain of all wheat cultivars tested was ferulic acid, with values ranging from 85.3% to 89.3% of the total content of PA.

In the present study, in addition to ferulic acid, we also identified sinapic, caffeic, syringic, *p*-coumaric, and *o*-coumaric acid, as well as isomers of di-ferulic acid (II, III, IV, V, and VI), in the grain of four genotypes of winter wheat (Table 4). Zuchowski et al. [33] and Hefni et al. [34] reported a similar identification of PA in wheat grain, but they additionally identified vanillic acid, which was not confirmed in the present study.

In the samples of *T. aestivum* ssp. *vulgare* (‘Tonacja’) wheat grain harvested in 2012, the higher total content of PA, amounting to 1188.75 mg 100 g^−1^ DW, was obtained in the grain from the medium level of cultivation technology (A), while a lower value was recorded in the grain from the high level of technology (B), amounting to 916.23 mg 100 g^−1^ DW. In 2012, the range of individual PA concentrations in this cultivar’s grain varied, ranging from 3.33 mg 100 g^−1^ DW for 3-OH-Benzoic acid to 633.9 mg 100 g^−1^ DW for ferulic acid. The content of PA recorded in the grain of this wheat cultivar in 2013 was higher in the case of cultivation technology A, i.e., the medium level of cultivation technology (Table 4). Similar results were obtained by Stuper-Szablewska et al. [35], according to whom biotic and abiotic stressors can activate natural defence mechanisms in some species of wheat. Stuper-Szablewska et al. [35] analysed changes in the quantitative profile of 12 PAs, the sum of bound PA, and the sum of free PA in the grains of 23 genotypes of winter wheat exposed to stress (artificial inoculation with fungal spores of the genus *Fusarium* or chemical protection). In that study, higher concentrations of bound PA were noted in the inoculated samples.

In contrast, the chemically protected samples did not differ significantly regarding this trait between genotypes or years. Similar findings were reported by Yilmaz et al. [14], who obtained a higher total PA concentration in wild wheat grain than domesticated wheat species. The findings of Zuchowski et al. [34] are in agreement with those of Yilmaz et al. [14] and Stuper-Szablewska et al. [35], as the total PA content in their research, was also higher in wheat grown in an organic system than in wheat grown conventionally. The studies cited above confirm the results of our study, in which the content of PA was higher in the grain of wheat grown using the medium cultivation technology. This suggests that the medium cultivation technology may be more conducive to the production of wheat with a higher PA content, which has potential health benefits for consumers.

We noted similar total contents of PA for the two levels of cultivation technology in the case of spelt *T. aestivum* ssp. *spelta* (‘Schwabenkorn’ cultivar). The total PA content in the grain of this species harvested in 2012 was 998.74 mg of 100 g^−1^ DW and 978.71 mg of 100 g^−1^ DW for cultivation technologies A and B, respectively. The higher total content of PA was also obtained in the grain of ‘Schwabenkorn’ spelt using the medium level of cultivation technology (A). In comparison, a lower content was obtained in the grain of ‘Schwabenkorn’ spelt from the high level of cultivation technology (B) (Table 4). Similar relationships with the level of cultivation technology have been reported by Gawlik-Dziki et al. [36].

In the case of durum wheat *T. turgidum* ssp. *durum* (‘Komnata’) and *T. monococcum* PL 5003, we observed a higher total content of PA in the grain from the high level of cultivation technology. This relationship for these cultivars was also observed in the grains harvested in 2012 and 2013. The total content of PA in the samples of ‘Komnata’ wheat harvested in 2012 from the medium level of cultivation technology was 1026.69 mg of 100 g^−1^ DW, with results ranging from 3.96 mg of 100 g^−1^ DW (syringic acid) to 465.2 mg of 100 g^−1^ DW (ferulic acid). For the grain harvested from the high level of cultivation technology in the same year, the mean total content of PA was 1372.81 mg of 100 g^−1^ DW, in a range from 3.10 mg of 100 g^−1^ DW (*o*-coumaric acid) to 731.5 mg of 100 g^−1^ DW (ferulic acid). This pattern was similar for the wheat cultivars harvested in 2013, with the total content of polyphenolic compounds for the winter wheat of the species *T. monococcum* (PL 5003) grown at the medium level of cultivation technology (A) amounting to 903.21 mg of 100 g^−1^ DW, while, for the high level of cultivation technology (B), it was 1254.01 mg of 100 g^−1^ DW. The total content of PA in the grain of the same cultivar in 2013 was nearly double the amount from 2012 for both levels of cultivation technology, with the highest content also recorded in the grains of wheat from cultivation technology B.

Contrasting results are presented by Stumpf et al. [37], as the use of cultivation technology with a higher nitrogen application did not affect the total content of PA. Tian et al. [38] suggest that the application of nitrogen fertilisers need not be the main factor influencing the level of PA in wheat grain. According to Lopes et al. [39] and Costanzo et al. [40], the total content of PA in different wheat genotypes may be highly dependent on climate conditions, the level of cultivation technology, and the genetic traits of the plants. Those authors found a varied content of PA in three wheat species (einkorn: *T. monococcum* L., emmer: *T. dicoccon* Schrank, and spelt: *T. spelta* L.) grown in organic conditions for three consecutive years. This has also been observed in a few other studies, in which einkorn wheat grain and flour were compared with spelt and emmer [41,42]. Significant differences in the PA content in wheat between years have also been reported by Lachman et al. [43] and Buczek et al. [44]. A higher total polyphenolic concentration may be due to lower rainfall and higher temperatures during the ripening stages of cereals. This is not confirmed by the results of the present study, in which all tested genotypes of winter wheat had higher levels of PA in 2013, when rainfall was more abundant, especially in May and June—the months of the critical stage for cereals. According to Skrajda et al. [45] and Lacko-Bartošová et al. [46], the content and composition of PA depend on multiple factors, such as the species and cultivar, and the cultivation conditions (sowing time, cultivation technology, fertilisation, plant protection strategy, location, and climate conditions). Mpofu et al. [25] showed that environmental factors had a more significant influence than genetic factors on the content of phenolic compounds. The researchers demonstrated that as much as 96% of the PA content variance was due to the environment × genotype interaction. Li et al. [47] and Shewry et al. [1], in their work for the HEALTHGRAIN project, determined differences in the content of PA depending on the cultivar. Among all genotypes, common bread wheat had the highest content of these compounds (up to 1171 μg/g), with substantial cultivar differences (>3.5-fold differences in content). This variation was the most minor in spelt (1.9-fold), with values in the 382–726 μg/g range. For the remaining wheat, the ranges were 536–1086 μg/g (durum), 449–816 μg/g (einkorn), and 508–1161 μg/g (emmer) [48]. Belobrajdic and Bird [49] reported a range of 200–900 μg/g total PA in wheat grain, which is supported by the present study.

### 3.3. Scaled Heat Map for the Grain of Four Genotypes of Winter Wheat in Terms of the Content of Phenolic Acid (PA)

**Figure 1:**
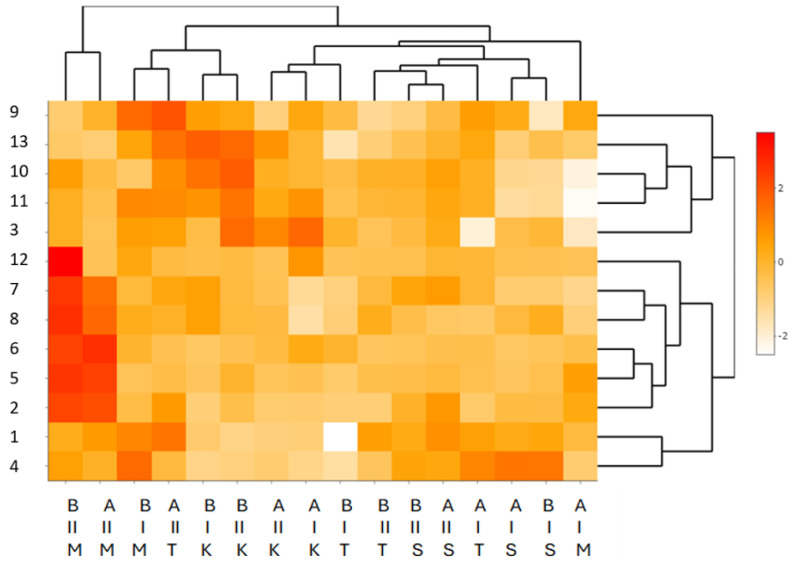
Scaled heat map for the grain of four genotypes of winter wheat in terms of the content of PAs. Explanations: Y-axis (Y) phenolic acids, PAs: 1—4-OH-benzoic acid. 2—caffeic acid. 3—OH-benzoic acid. 4—syringic acid. 5—*p*-coumaric acid. 6—*o*-coumaric acid. 7—ferulic acid. 8—sinapic acid. 9—di-ferulic acid isomer I. 10—di-ferulic acid isomer II. 11—di-ferulic acid isomer III. 12—di-ferulic acidisomer IV. 13—di-ferulic acid isomer V. X-axis, first row: Level of cultivation technology: A—medium level of cultivation technology. B—high level of cultivation technology. X-axis, second row: crop years: I—2011/2012. II—2012/2013. X-axis, third row: Genotype: M—*T. monococcum* PL 5003. T—*T. aestivum* ssp. *vulgare* ‘Tonacja’. S—*T. aestivum* ssp. *spelta* ‘Schwabenkorn’. K—*T. turgidum* ssp*. durum* ‘Komnata’.

The multidimensional exploration of the data based on a scaled heat map (Figure 1) for the content of PA in four genotypes of winter wheat (M—*T. monococcum* PL 500′. T—*T. aestivum* ssp*. vulgare* ‘Tonacja’. S—*T. aestivum* ssp. *spelta* ‘Schwabenkorn’. K—*T. turgidum* ssp. *durum* ‘Komnata’) in two growing seasons (2011/2012 [I] and 2012/2013 [II]) and at two levels of cultivation technology (A—medium level of cultivation technology, B—high level of cultivation technology) revealed interspecific differences. The clustering of data on the contents of PA revealed three clusters characterised by the similarity of these compounds. The first cluster was associated with the following PAs: 3-OH-benzoic acid, 9-di-ferulic acid isomer 1,10-di-ferulic acid isomer II, 11-di-ferulic acid isomer III, and 13-di-ferulic acid isomer V. The second cluster contained the following PAs: 12-di-ferulic acid isomer IV, 7-ferulic acid, 8-sinapic acid, 6-*o*-coumaric acid, 5-*p*-coumaric acid, and 2-caffeic acid. The remaining PAs (1-4-OH-benzoic acid. 4-syringic acid) formed the third cluster. The remaining structure of the data and their reduction were associated with the levels of cultivation technology, the years of cultivation, and the genotypes of winter wheat. The scaling of the data resulted in five distinct clusters, of which the first (A—the medium level of cultivation technology, B—the high level of cultivation technology, II—growing season 2012/2013, and wheat genotype *T. monococcum* PL 5003) was associated with the highest content of PAs. The second cluster was associated with a medium content of the compounds, while the third and fourth clusters were characterised by a low content of PAs. The fifth cluster was associated with very low levels of the compounds teste

## 4. Conclusions

The study of winter wheat genotypes showed that the total content of identified PAs varied and depended on the genotype of the cultivars tested, the level of cultivation technology, and the weather conditions during the wheat growing season.

The highest content of PAs was found in the PL 5003 cultivar of *Triticum monococcum*, which might predispose this ‘old’, somewhat forgotten species for use in the production of functional food. The response of each of the genotypes to the levels of cultivation technology applied varied. Common wheat and spelt responded with a decrease in the content of acids when tested at the higher level of cultivation technology. In contrast, the content of durum wheat and einkorn increased. A pronounced reaction to weather conditions was observed as well. Three of the four genotypes had a higher content of PA in the year when rainfall was more abundant. The results provide a basis for selecting the genotypes tested with a high content of biologically active compounds for food processing. The relationships demonstrated between the content of individual PAs, the level of cultivation technology, and weather conditions can be applied to introduce adjustments to these species’ cultivation technology. Changes in technology could affect, for example, differentiation in terms of cultivation intensification. The studies that were conducted showed that reducing fertilisation and plant protection has a beneficial effect on the content of bioactive components in some genotypes. Further research is needed to determine which wheat cultivars would enable food production to increase its nutritional value. An inspiration for further research may also be the consideration of the economic efficiency of the applied level of agricultural technology in relation to the quality (price) of the raw materials obtained.

## Figures and Tables

**Table 1:** *Triticum* species and cultivars tested in this study.

Wheat Species	Latin Name	Cultivar
Common wheat	*T. aestivum* ssp*. vulgare*	‘Tonacja’
Spelt	*T. aestivum* ssp. *spelta*	‘Schwabenkorn’
Durum wheat	*T. turgidum* ssp. *durum*	‘Komnata’
Einkorn	*T. monococcum*	PL 5003

**Table 2:** According to the Meteorological Observatory at Felin, rainfall and air temperatures in 2011–2013 compared to the long-term averages (1951–2010) are presented.

Year	Month											
	Sep	Oct	Nov	Dec	Jan	Feb	Mar	Apr	May	Jun	Jul	Aug
	Rainfall in mm											
2011/2012	5.4	28.5	1.0	34.5	33.6	22.1	28.6	34.0	56.3	62.8	52.3	37.6
2012/2013	35.5	88.8	29.8	28.8	57.7	28.5	60.8	51.1	101.6	105.9	126.1	17.8
Mean for 1951–2010	53.7	40.1	38.2	31.4	23.4	25.8	28.0	39.0	60.7	65.9	82.0	70.7
**Year**	**Air temperature in °C**											
2011/2012	15.2	8.0	2.4	2.0	−1.8	−7.1	4.3	9.5	15.0	17.3	21.5	19.2
2012/2013	15.0	8.1	5.5	−3.8	−3.8	−1.0	−2.4	8.1	15.3	18.5	19.2	19.2
Mean for 1951–2010	12.6	7.6	2.6	−1.6	−3.7	−2.8	1.0	7.4	13.0	16.3	18.0	17.2

**Table 3:** Separation parameters of phenolic acid standards by the UPLC/MS method.

No.	Retention Time	[M − H]~	Fragment Ion	Λ_max_	Quality Transition	Collision Energy	Identification
	[min]	[*m*/*z*]	[*m*/*z*]	[nm]	[*m*/*z*]	[eV]	
1.	7.47	137	93	230	137→93	10	4-OH-Benzoic acid benzoic
2.	9.75	179	161, 135	320	179→163	20	Caffeic acid
3.	10.22	137	93	223	137→93	20	3-OH-Benzoic acid
4.	10.58	197	179, 135	277	197→135	30	Syringic acid
5.	11.24	163	119	312	163→119	30	*p*-Coumaric acid
6.	11.92	163	119	312	163→119	30	*o*-Coumaricacid
7.	12.2	193	149, 134	322	193→134	30	Ferulic acid
8.	12.4	223	179, 149	320	223→179	30	Sinapic acid
9.	12.93	385	297	323	385→297	30	di-Ferulic acid (isomer I)
10.	13.25	385	245	320	385→245	30	di-Ferulic acid (isomer II)
11.	14.47	385	319	322	385→193	30	di-Ferulic acid (isomer III)
12.	15.25	385	193	325	385→193	30	di-Ferulic acid (isomer IV)
13.	15.43	385	293	322	385→193	30	di-Ferulic acid (isomer V)

**Table 4:** Content of PA in the grain of winter wheat in various systems of cultivation technology in mg 100 g^−1^ DM.

Cultivars	Crop Years	Cultivation Technology	4-OH Benzoic Acid	Caffeic Acid	3-OH-Benzoic Acid	Syringic Acid	*p*-Coumaric Acid	*o*-Cumaric Acid	Ferulic Acid	Sinapic Acid	di-Frulic Acid Isomer I	di-Ferulic Acid Isomer II	di-Ferulic Acid Isomer III	di-Ferulic Acid Isomer IV	di-Ferulic Acid Isomer V	Sum
*T. aestivum* ssp*. vulgare* ‘Tonacja’	2012	A	63.88 ± 1.76	14.49 ± 0.84 b,c	3.33 ± 0.09 b,c	9.79 ± 0.4 b,c	48.20 ± 2.98 b,c	6.60 ± 0.24 b,c	633.9 ± 2.4 b,c	79.85 ± 0.83 b,c	21.74 ± 1.32 a,c	159.7 ± 8.76 b,c	111.0 ± 8.28 b,c	13.15 ± 0.43 b,c	23.06 ± 0.76 b,c	1188.69
		B	21.91 ± 1.21	11.64 ± 0.33 a	9.29 ± 0.26 a,d	3.22 ± 0.08	23.13 ± 2.83 a,d	10.70 ± 0.88 a,d	505.6 ± 2.05 a,d	74.37 ± 0.51 a,d	14.17 ± 0.73 a,d	142.2 ± 5.3 a,d	86.7 ± 4.63 a,d	3.72 ± 0.24 a,d	9.55 ± 0.28 a,d	916.21
	2013	A	75.33 ± 1.12 a,d	43.23 ± 0.58 a,d	10.98 ± 0.34 a,d	6.40 ± 0.31 a,d	55.69 ± 2.2 a,d	5.55 ± 0.54 a,d	704.2 ± 2.6 a,d	103.7 ± 1.45 a,d	33.10 ± 1.28 a,d	191.9 ± 4.17 a,d	146.4 ± 3.8 a,d	9.28 ± 0.39 a,d	30.75 ± 0.33 a,d	1416.51
		B	64.63 ± 1.36 b,c	11.77 ± 0.43 a	7.81 ± 0.42 b,c	5.38 ± 0.31 b,c	52.98 ± 2.34 b,c	3.88 ± 0.63 b,c	618.4 ± 2.3 b,c	108.7 ± 1.7 b,c	6.23 ± 0.23 b,c	158.3 ± 1.65 b,c	100.9 ± 1.26 b,c	4.80 ± 0.52 b,c	14.32 ± 0.68 b,c	1158.1
*T. aestivum* ssp. *spelta* ‘Schwabenkorn’	2012	A	60.34 ± 1.08 b,c	23.79 ± 1.26 c	8.30 ± 0.17 c	10.61 ± 0.60 c	34.20 ± 2.02 b,c	3.30 ± 0.23 b,c	529.2 ± 2.6 c	95.28 ± 1.03 b,c	18.81 ± 0.27	108.8 ± 1.21 b,c	45.8 ± 1.3 b,c	5.30 ± 0.21 b,c	14.51 ± 0.64 b,c	998.74
		B	62.48 ± 1.17 a,d	23.49 ± 1.35 d	8.97 ± 0.44 d	10.60 ± 0.60 d	49.68 ± 1.36 a,d	4.38 ± 0.46 a	529.8 ± 1.47 d	108.0 ± 1.3 a,d	1.93 ± 0.16 a,d	106.3 ± 1.67 a,d	50.9 ± 0.86 a,d	4.33 ± 0.15 a,d	17.85 ± 0.53 a	978.71
	2013	A	68.33 ± 1.5 a,d	44.13 ± 0.77 a,d	10.14 ± 0.31 a,d	7.93 ± 0.33 a	63.50 ± 2.36 a,d	6.32 ± 0.37 a,d	744.2 ± 2.5 a,d	82.27 ± 1.86 a,d	14.04 ± 0.93 a,d	176.9 ± 1.7 a,d	120.7 ± 1.8 a,d	14.48 ± 0.28 a,d	20.40 ± 0.48 a,d	1373.34
		B	60.52 ± 0.88 b,c	30.06 ± 0.45 b,c	8.60 ± 0.12 c	8.24 ± 0.83 b	53.63 ± 0.8 b,c	4.65 ± 0.23 c	718.8 ± 2.7 b,c	91.09 ± 0.76 b,c	8.36 ± 0.85 b,c	159.4 ± 1.58 b.c	102.2 ± 0.94 b,c	5.96 ± 0.17 b,c	17.44 ± 0.31 c	1268.95
*T. turgidum* ssp. *durum* ‘Komnata’	2012	A	44.02 ± 0.74	13.75 ± 0.68 b	14.06 ± 0.36 b,c	3.96 ± 0.23	46.27 ± 0.79 b,c	14.38 ± 0.64 b,c	465.2 ± 1.7 b,c	56.52 ± 0.8 b,c	19.67 ± 0.48 b,c	151.9 ± 1.6 b,c	139.5 ± 1.65 c	37.42 ± 0.78 b,c	19.99 ± 0.25 b,c	1026.64
		B	45.65 ± 1.45	11.39 ± 0.65 a,d	8.40 ± 0.26 a,d	4.02 ± 0.47	35.48 ± 0.34 a,d	3.10 ± 0.28 a,d	731.5 ± 2.4 a,d	119.3 ± 1.4 a,d	21.41 ± 0.31 a,d	212.0 ± 1.65 a,d	140.2 ± 1.7 d	7.14 ± 0.37 a,d	33.10 ± 0.36 a,d	1372.69
	2013	A	43.29 ± 1.86	13.48 ± 0.34 d	12.34 ± 0.25 a.d	4.80 ± 0.48	38.47 ± 0.71 a,d	7.69 ± 0.23 a,d	579.7 ± 2.3 a,d	94.3 ± 0.9 a,d	8.19 ± 0.32 a,d	161.7 ± 1.57 a,d	118.6 ± 1.68 a,d	3.40 ± 0.15 a,d	26.24 ± 0.24 a,d	1112.2
		B	41.67 ± 1.03	21.80 ± 0.32 b,c	13.99 ± 0.26 b,c	4.35 ± 0.25	79.99 ± 3.28 b,c	5.49 ± 0.24 b,c	613.7 ± 2.6 b,c	96.58 ± 0.45 b,c	19.44 ± 0.35 b,c	229.0 ± 1.8 b,c	163.3 ± 1.66 b,c	9.31 ± 0.13 b,c	31.75 ± 0.26 b,c	1330.37
*T. monococcum* ‘PL 5003′	2012	A	53.24 ± 1.22	36.08 ± 0.29 b,c	4.14 ± 0.13 b,c	4.81 ± 0.27 b,c	130.1 ± 1.68 b,c	6.60 ± 0.28 b,c	482.2 ± 1.84 b,c	73.50 ± 0.38 b,c	19.49 ± 0.41 b,c	70.48 ± 0.36 b,c	3.45 ± 0.14 b,c	3.38 ± 0.17 b	15.53 ± 0.16 b.c	903.21
		B	71.17 ± 1.43	23.08 ± 0.65 a,d	11.07 ± 0.35 a,d	11.19 ± 0.28 a,d	40.7 ± 0.36 a,d	10.70 ± 0.23 a,d	621.4 ± 2.67 a,d	108.73 ± 1.63 a,d	29.89 ± 0.37 a,d	126.7 ± 1.12 a,d	148.4 ± 1.48 a,d	27.19 ± 0.21 a,d	23.91 ± 0.24 a,d	1254.13
	2013	A	66.07 ± 1.12	71.31 ± 1.05 a,d	7.78 ± 0.35 a,d	7.21 ± 0.43 a,d	268.5 ± 1.87 a,d	42.72 ± 0.68 a,d	868.3 ± 2.86 a,d	152.7 ± 1.43 a,d	16.53 ± 0.26 a,d	144.8 ± 1.17 a,d	87.82 ± 1.11 a,d	3.51 ± 0.14 d	14.58 ± 0.18 a,d	1751.83
		B	59.64 ± 1.07	74.88 ± 1.15 b,c	9.73 ± 0.45 b,c	8.49 ± 0.35 b,c	286.1 ± 1.43 b,c	38.28 ± 0.36 b,c	1017 ± 3.44 b,c	185.2 ± 2.24 b,c	9.79 ± 0.21 b,c	178.3 ± 1.24 b,c	111.5 ± 1.38 b,c	111.5 ± 1.38 b,c	15.65 ± 0.16 b,c	2106.06

A—medium level of cultivation technology; B—high level of cultivation technology. The letters a–d indicate statistically significant differences in the content of individual phenolic acids within one cultivar. between the means tested by ANOVA in Tukey’s post-hoc test at *p* < 0.05.

## Data Availability

The original contributions presented in this study are included in the article. Further inquiries can be directed to the corresponding author.
